# Synergistic Zn/Al Co-Doping and Sodium Enrichment Enable Reversible Phase Transitions in High-Performance Layered Sodium Cathodes

**DOI:** 10.3390/molecules30234628

**Published:** 2025-12-02

**Authors:** Yaru Qin, Tingfei Yang, Na Chen, Jiale Li, Anqi Li, Yu Miao, Chenglong Shi, Jianmin Ma, Xue Qin

**Affiliations:** 1 School of Chemistry and Materials Science, Qinghai Minzu University, Xining 810007, China; 2 Key Laboratory of Resource Chemistry and Eco-Environmental Protection on Tibetan Plateau, State Ethnic Affairs Commission, Qinghai Minzu University, Xining 810007, China; 3 Department of Chemistry, School of Science, Tianjin University, Tianjin 300072, China

**Keywords:** sodium-ion batteries, P2-type cathodes, co-doping, Na enrichment, reversible phase transitions

## Abstract

Layered transition-metal oxides are among the most promising sodium-ion battery cathodes owing to their high specific capacities and structurally tunable frameworks. However, the prototypical P2-Na_0.67_Ni_0.33_Mn_0.67_O_2_ (NM) undergoes an irreversible P2 → O2 phase transition at high voltages, accompanied by severe lattice strain and capacity fade, which hinders practical deployment. Here, we propose a cooperative regulation strategy that couples Zn/Al co-doping with Na enrichment, and successfully synthesize P2-Na_0.80_Ni_0.14_Zn_0.14_Mn_0.58_Al_0.14_O_2_ (NMZA-N14). The optimized NMZA-N14 delivers an initial discharge capacity of 125 mAh g^−1^ at 0.1C and demonstrates exceptional cycling and rate performance, retaining 98.6% of its capacity after 100 cycles at 0.2C and 93.6% after 200 cycles at 1C. Kinetic analyses indicate a higher Na^+^ diffusion coefficient and a lower charge-transfer resistance in NMZA-N14, evidencing substantially accelerated ion transport. In situ X-ray diffraction further reveals a reversible P2 → OP4 phase transition in the high-voltage regime with a unit-cell volume change of only ~2.27%, thereby avoiding the irreversible structural degradation observed in NM. This synergistic modulation markedly enhances structural stability and electrochemical kinetics, providing a viable pathway for the rational design of high-performance sodium-ion battery cathodes.

## 1. Introduction

Sodium-ion batteries (SIBs) are regarded as a promising alternative to lithium-ion systems owing to the abundance of sodium, lower cost, and favorable safety, showing broad prospects for large-scale energy storage and related applications [1,2]. Among various cathode candidates, layered transition-metal oxides have drawn extensive attention because of their high specific capacities and facile synthesis [3,4]. Layered oxides with the general formula Na_x_TMO_2_ (TM = transition metal) primarily adopt P2- and O3-type stacking, and represent one of the most application-ready classes of cathode materials [5,6]. The prototypical P2-Na_2/3_Ni_1/3_Mn_2/3_O_2_, featuring a reversible Ni^2+^/Ni^4+^ redox couple, delivers a relatively high theoretical capacity (~173 mAh g^−1^) at a moderate operating voltage (~3.5 V), and has therefore garnered considerable interest [7]. Nevertheless, Na^+^/vacancy ordering, complex phase evolutions, and lattice distortions commonly arise during cycling, leading to rapid capacity decay [8]. In particular, at high voltages (>4.2 V), P2-Na_0.67_Ni_0.33_Mn_0.67_O_2_ often undergoes an irreversible P2 → O2 transition accompanied by TM-slab gliding and pronounced volume change, which severely compromises cycling stability [9,10]. Consequently, rational structural-modulation strategies for this family of materials have become a central research focus.

In recent years, a variety of modification strategies have been advanced to improve the overall electrochemical performance of layered oxides [11]. Introducing heteroatoms into the transition-metal (TM) slabs is a widely adopted and effective approach: dopants such as Cu [12], Fe [13], and Mg [14] can stabilize the lattice, modulate charge distribution, and enhance cycling durability. For example, Chen et al. [15] realized cooperative regulation of Na^+^ transport kinetics and high-voltage phase evolution via Mg^2+^/F^−^ co-doping, leading to a markedly improved capacity retention after 750 cycles at 1000 mA g^−1^. Zhang et al. [16] showed that Al substitution effectively suppresses the Jahn-Teller distortion of Mn^3+^, thereby eliminating the detrimental P2 → P2′ transition and significantly extending cycle life; the electrode retained 92.8% of its capacity after 200 cycles at 1C within 2.0–4.0 V. Wang et al. [17] further found that high-level Zn doping strongly suppresses the P2 → O2 transition, constraining the volume change to ~1.0% during cycling and affording excellent rate capability and cycling stability. In addition, inserting “pillar ions” into the alkali-metal (AM) layers has been shown to help maintain the layered framework. For instance, Wu et al. [18] reported a P2-type Li dual-site-substituted cathode in which Li^+^ occupies both TM and AM positions, simultaneously widening the voltage window and delivering near-zero-strain behavior. Taken together, the synergistic regulation of both TM and AM layers is pivotal to structural stability and electrochemical performance in layered cathodes.

Building on this context, we propose a synergistic strategy that couples transition-metal (TM) slab substitution with Na-layer enrichment to enhance P2-type layered oxides. Zn^2+^ and Al^3+^ are introduced into the TM slabs to stabilize the TM-O framework and mitigate Jahn-Teller distortion; meanwhile, the Na content is increased from 0.67 to 0.80 to provide additional ionic support in the high-voltage regime, thereby strengthening electrostatic shielding and suppressing interlayer gliding. Following this design, a Na-enriched P2 phase, Na_0.80_Ni_0.14_Zn_0.14_Mn_0.58_Al_0.14_O_2_, was synthesized and subjected to structural characterization, electrochemical-kinetics analyses, and in situ X-ray diffraction. The results reveal pronounced improvements in cycling stability, rate capability, and phase-transition reversibility, validating the effectiveness of synergistically modulating the TM and Na layers for optimizing layered sodium-ion battery cathodes.

## 2. Results and Discussion

### 2.1. Crystal Structures

**Figure 1:**
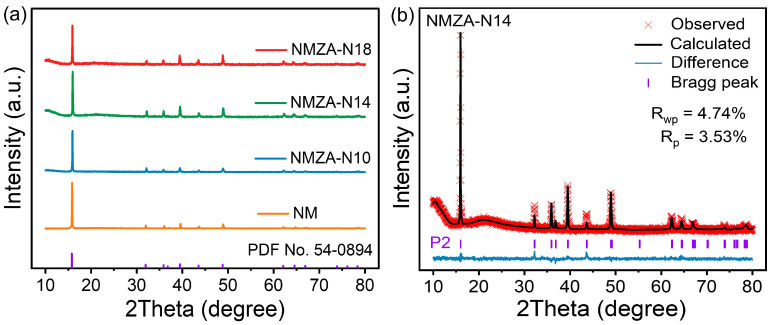
XRD patterns of the NM and NMZA series (**a**), and Rietveld refinement profile of NMZA-N14 (**b**).

To elucidate the crystal structures of NM, NMZA-N10, NMZA-N14, and NMZA-N18, powder XRD was performed (Figure 1a). All patterns display sharp reflections with high signal-to-noise ratios, indicative of good crystallinity. The dominant peaks match those of a P2-type layered framework (space group P6_3_/mmc, PDF No. 54-0894). Characteristic O3 reflections near 2θ ≈ 16.58° (O3-(003)) and 33.57° (O3-(006)) are absent, and no spinel/rock-salt impurities are detected, confirming that all four cathodes crystallize in a single P2 phase. Rietveld refinements were carried out using GSAS-II (Version 5455) (Figure 1b and Figure S1). The refined lattice parameters and R-factors for all samples are summarized in Table S1. Low residuals (R_p_ ≈ 3.1–5.0%, R_wp_ ≈ 4.6–6.4%) and flat difference curves attest to excellent agreement between the model and experimental data. The refined structures adopt a hexagonal P6_3_/mmc symmetry with in-plane lattice parameters a = b ≈ 2.87–2.90 Å. The undoped NM exhibits a c-axis parameter of 11.184 Å, whereas that of NMZA-N14 contracts slightly to 11.135 Å. This contraction primarily arises from Al^3+^ substitution (VI coordination radius 0.535 Å), which shortens TM-O bonds and thins the MO_6_ octahedral slabs [19]. Concurrently, Zn^2+^ incorporation effectively mitigates the P2 → O2 transition and high-voltage structural distortions [20]. In addition, a higher Na content optimizes the interlayer electrostatic environment, enhancing the shielding and mechanical support of the oxygen layers by Na^+^, thereby maintaining a reasonable interslab spacing and suppressing layer gliding [21]. Collectively, these synergistic effects drive a modest c-axis contraction and reinforce the layered framework. Structurally, a moderate reduction of c stabilizes oxygen stacking, suppressing gliding and irreversible phase transitions during cycling, which improves bulk structural stability and provides a robust basis for reversible capacity retention and rate capability.

**Figure 2:**
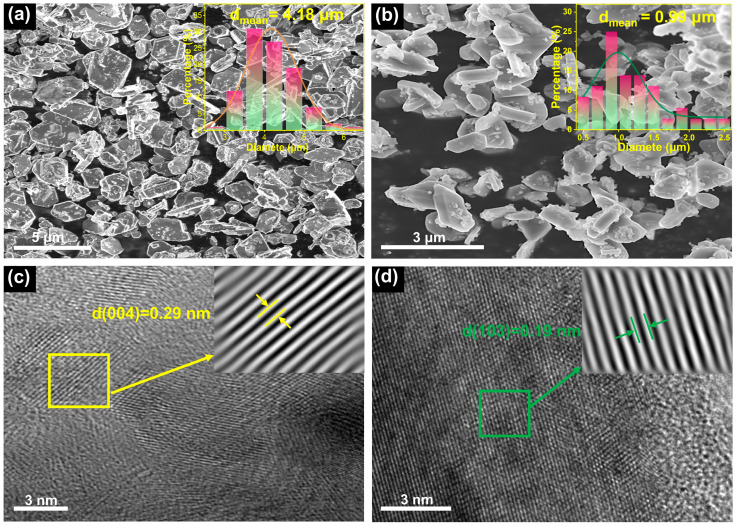
SEM micrographs of (**a**) NM and (**b**) NMZA-N14 with particle-size distribution insets; HRTEM images of (**c**) NM and (**d**) NMZA-N14.

As shown in Figure 2a,b, both NM and NMZA-N14 exhibit the characteristic plate-like polygonal morphology of layered sodium-ion cathodes. However, their particle-size distributions differ markedly: for NM, particle sizes are mainly distributed between 2–7 μm with an average of ~4.18 μm, whereas NMZA-N14 shows significantly smaller particles of 0.5–2.5 μm with an average of ~0.99 μm. Reducing the particle size shortens the solid-state diffusion path for Na^+^ and lowers interfacial polarization, thereby improving electrochemical kinetics. The particle-size refinement observed in NMZA-N14 may be associated with the influence of Zn^2+^/Al^3+^ substitution on crystal-growth behavior during calcination. Such aliovalent doping can modify the local chemical environment and alter the growth kinetics, which may increase the nucleation probability and suppress excessive grain coarsening. Collectively, these effects are likely to facilitate the formation of finer plate-like particles [22,23]. High-resolution transmission electron microscopy (HRTEM) further resolves the local structural features (Figure 2c,d). After fast Fourier transform (FFT) and inverse-FFT filtering of selected regions, well-defined lattice fringes are observed, d(004) = 0.29 nm for NM and d(103) = 0.19 nm for NMZA-N14, both consistent with a P2-type layered framework [24], indicating that Zn/Al co-doping preserves the layered backbone with no discernible secondary phases in the probed areas. Moreover, EDS elemental mapping (Figure S2) reveals homogeneous distributions of Na, O, Ni, Mn, Zn, and Al within NMZA-N14 particles, confirming uniform incorporation of Zn and Al into the NMZA-N14 lattice.

**Figure 3:**
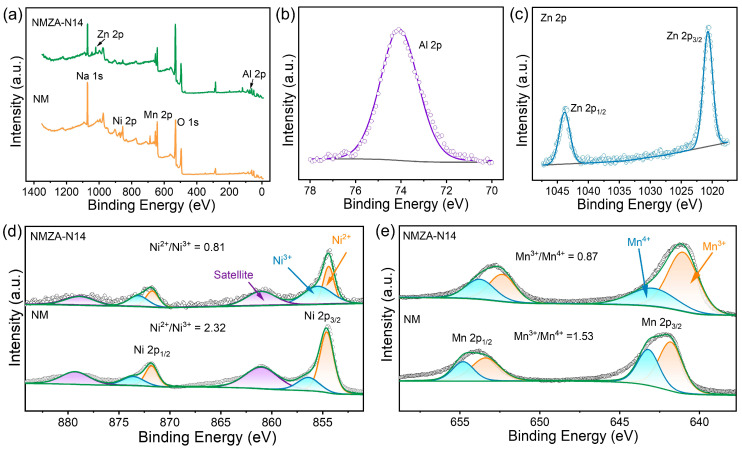
(**a**) XPS survey spectra of NM and NMZA-N14; (**b**) XPS spectrum of Al 2p; (**c**) XPS spectrum of Zn 2p; (**d**) Ni 2p spectra of NM and NMZA-N14; (**e**) Mn 2p spectra of NM and NMZA-N14.

XPS was employed to probe the chemical composition and near-surface oxidation states of the samples. As shown in Figure 3a, distinct Zn 2p and Al 2p signals are present in the survey spectrum of NMZA-N14 but are absent in the undoped NM, confirming the successful incorporation of Zn and Al. Further analysis indicates that the Al 2p feature appears at ~74 eV, characteristic of Al^3+^ (Figure 3b). The Zn 2p_3/2_ and Zn 2p_1/2_ components are located at ~1021 and ~1044 eV, respectively, consistent with Zn^2+^ (Figure 3c), indicating that it is present in a stable divalent state in the material. High-resolution spectra of the transition metals (Figure 3d,e) further resolve changes in the valence-state distribution. The Ni 2p region shows main peaks at ~854.6 eV (Ni 2p_3/2_) and ~871 eV (Ni 2p_1/2_), accompanied by characteristic shake-up satellites. Deconvolution reveals the coexistence of Ni^2+^ and Ni^3+^ in both NM and NMZA-N14; however, the Ni^2+^/Ni^3+^ ratio decreases from 2.32 in NM to 0.81 in NMZA-N14, indicating a pronounced Zn/Al co-doping-induced oxidation from Ni^2+^ to Ni^3+^. Prior studies have shown that a higher fraction of Ni^3+^ enhances charge compensation and facilitates reversible Na^+^ (de)intercalation, thereby improving electrode kinetics [25]. For Mn, peaks at ~641.2 eV (Mn 2p_3/2_) and ~653.0 eV (Mn 2p_1/2_) are observed (Figure 3e). Fitting indicates the presence of both Mn^3+^ and Mn^4+^ in both samples. The Mn^3+^/Mn^4+^ ratio drops from 1.53 in NM to 0.87 in NMZA-N14, with Mn^4+^ becoming prevalent. As reported previously, excess Mn^3+^ induces Jahn-Teller distortions and destabilizes MnO_6_ octahedra, accelerating capacity fade, whereas Mn^4+^ remains stable within the operating window and effectively suppresses adverse structural evolution [26]. The Mn 3s spectra of NM and NMZA-N14 are shown in Figure S3. Standard peak deconvolution of the Mn 3s region yields exchange-splitting values (ΔEs) of 4.8 eV for NM and 4.6 eV for NMZA-14. To further verify the Mn valence state, we calculated the average oxidation state (AOS) using the established correlation AOS = 8.956–1.126 ΔEs [27,28]. The resulting AOS values are +3.55 for NM and +3.78 for NMZA-14, clearly indicating that Zn/Al co-doping increases the fraction of Mn^4+^ while reducing the amount of Jahn–Teller-active Mn^3+^. Consequently, the Mn valence shift toward a higher oxidation state upon doping mitigates lattice distortion and enhances cycling stability. Collectively, Zn/Al co-doping enhances the Ni^3+^ content for faster ion transport and enriches Mn^4+^ to strengthen the structure, jointly improving the electronic and structural stability and thereby boosting rate and cycling performance.

### 2.2. Electrochemical Performance

**Figure 4:**
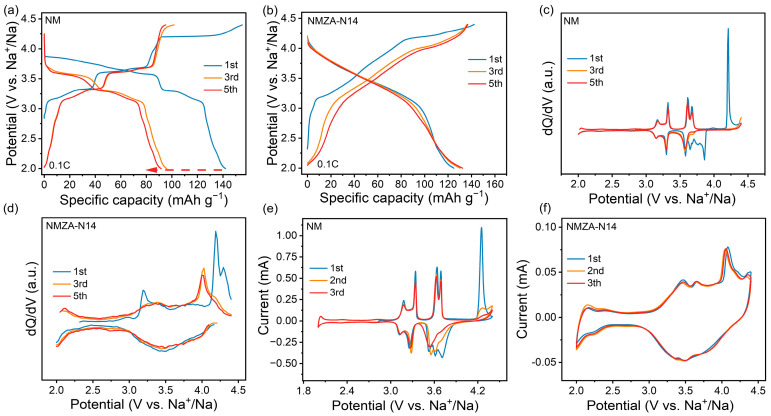
Charge-discharge profiles of (**a**) NM and (**b**) NMZA-N14; differential-capacity (dQ/dV) plots of (**c**) NM and (**d**) NMZA-N14; cyclic voltammetry (CV) profiles of (**e**) NM and (**f**) NMZA-N14.

Electrochemical performance was evaluated at 0.1C within 2.0–4.4 V. As shown in Figure 4a, the undoped NM delivers a first-cycle discharge capacity of 141.9 mAh g^−1^ but rapidly declines to 91.6 mAh g^−1^ by the 5th cycle, corresponding to an average per-cycle loss of ~12.6 mAh g^−1^. This pronounced fade indicates severe, irreversible structural evolution during early cycling. The differential capacity (dQ/dV) curves (Figure 4c) further clarify the reaction mechanism: multiple weak peaks between 2.0–3.0 V are attributable to the Mn^2+^/Mn^3+^/Mn^4+^ redox processes; two plateaus over 3.0–3.7 V arise from Na^+^/vacancy-ordering transitions; and a strong, rapidly vanishing redox peak in 4.0–4.4 V is consistent with the high-voltage phase transition characteristic of P2 layered oxides and commonly associated with P2 → O2 interlayer gliding [29]. Such an irreversible transition induces lattice distortion and capacity loss, thereby limiting the cycling stability of NM.

In contrast, NMZA-N14 exhibits distinctly different electrochemical behavior (Figure 4b). Its first-cycle discharge capacity is 125 mAh g^−1^, but it increases to 132.4 mAh g^−1^ by the 5th cycle, indicative of a typical first-cycle activation effect. As cycling proceeds, the charge–discharge curves progressively smoothen within 3.0–4.0 V: the 5th-cycle profile is the flattest, followed by the 3rd, whereas the 1st cycle shows a clear voltage inflection near ~3.2 V. This evolution suggests that initial Na^+^/vacancy ordering is gradually disrupted toward a more disordered state, enabling more reversible Na^+^ (de)intercalation and thereby improving capacity reversibility. The corresponding differential-capacity (dQ/dV) curves (Figure 4d) corroborate this trend: a prominent oxidation peak appears near ~3.2 V during the first charge but fades by the 3rd and 5th cycles, reflecting the progressive increase in Na^+^/vacancy disorder. Notably, NMZA-N14 retains a main oxidation feature near ~4.3 V upon de-sodiation, but its intensity and position differ from those of NM, indicating that Zn/Al co-doping together with Na enrichment modifies the high-voltage phase-transition pathway, rendering it milder and more reversible. More importantly, no pronounced Mn-related peaks emerge in the 2.0–3.0 V region for NMZA-N14, implying a substantial suppression of the Mn^3+^/Mn^4+^ redox at low voltage. This observation aligns with the XPS results, namely that Mn is predominantly Mn^4+^ after Zn/Al doping and remains valence-stable during cycling, thereby avoiding Mn^3+^-induced Jahn-Teller distortions and markedly enhancing structural stability [30]. Comparison across samples with different Na contents further substantiates this trend (Figure S4). The Na-enriched NMZA-N10 and NMZA-N14 exhibit progressively smoother voltage profiles upon cycling, reflecting the gradual disordering of Na^+^/vacancy ordering; by contrast, the stoichiometric-Na NM and NMZA-N18 show no pronounced activation behavior. Notably, within 2.0–3.0 V, the voltage curve of NMZA-N18 is smoother than that of NM, indicating that even without Na enrichment, Zn/Al co-doping effectively suppresses the low-valence Mn redox activity. These observations demonstrate that Zn/Al co-doping regulates the electrochemical activity of the transition-metal layers not only in Na-rich compositions but also across different Na contents. In summary, NM undergoes rapid capacity decay due to the participation of Mn^3+^ and the irreversible high-voltage P2 → O2 transition. In NMZA-N14, Na enrichment produces an “ionic support effect” in the Na layers, helping maintain interlayer stability; concurrently, Zn/Al co-doping adjusts the transition-metal valence through charge compensation, increasing the fractions of Mn^4+^ and Ni^3+^, thereby weakening Jahn-Teller distortions and enhancing Na^+^ (de)intercalation kinetics. This dual-modulation strategy markedly improves kinetic reversibility and structural stability, yielding a wider solid-solution window, higher reversible capacity, and superior cycling and rate performance.

To further evaluate the effects of Zn/Al co-doping and Na enrichment on electrochemical performance, cyclic voltammetry (CV) measurements were conducted on NM and NMZA-N14. For the undoped NM, the CV curves within 2.0–4.4 V exhibit multiple distinct oxidation/reduction peaks, indicating multiphase transitions during Na^+^ (de)intercalation and implying poor cycling stability (Figure 4e). By contrast, the CV profiles of NMZA-N14 (Figure 4f) are relatively smooth with a more continuous current response, suggesting that the reactions proceed predominantly via solid-solution behavior rather than abrupt phase transitions [31]. In addition, the first three cycles for NMZA-N14 nearly overlap with negligible peak attenuation, reflecting excellent reaction reversibility, consistent with its superior stability during cycling.

**Figure 5:**
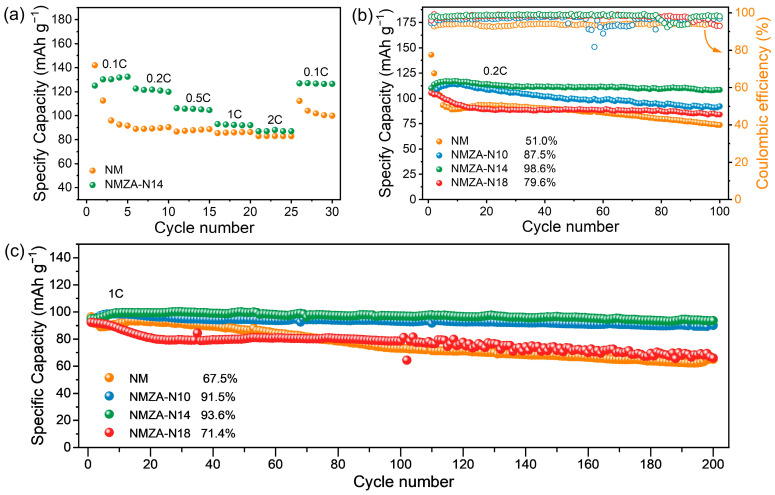
(**a**) Discharge capacity versus cycle number for NM and NMZA-N14 at various C-rates; (**b**) cycling performance of the NMZA series and NM at 0.2C; (**c**) cycling performance of the NMZA series and NM at 1C.

To assess rate capability, galvanostatic charge–discharge tests were carried out at various C-rates (Figure 5a). Although NMZA-N14 delivers a slightly lower first-cycle discharge capacity at 0.1C (125 mAh g^−1^) than NM (141.9 mAh g^−1^), it surpasses NM at subsequent rates. At 0.2C, 0.5C, 1C, and 2C, NMZA-N14 consistently maintains higher reversible capacities; notably, at 2C it still retains ~70.4% of its low-rate capacity. When the current density is returned from 2C to 0.1C, the capacity of NMZA-N14 recovers to 126.9 mAh g^−1^ with virtually no loss, further evidencing a structurally reversible host and fast kinetics.

Cycling-stability tests (Figure 5b,c) further highlight the advantages of NMZA-N14. After 100 cycles at 0.2C, NMZA-N14 retains a capacity of 108.5 mAh g^−1^, corresponding to a capacity retention of 98.6%, markedly outperforming NMZA-N10 (87.5%), NMZA-N18 (79.6%), and NM (51.0%). Even under a higher current of 1C for 200 cycles, NMZA-N14 still maintains 93.6% capacity retention, whereas NMZA-N10 and NMZA-N18 retain 91.5% and 71.4%, respectively, and NM falls to 67.5%. Meanwhile, its coulombic efficiency remains stably close to 100% throughout cycling (right y-axis of Figure 5b), further evidencing excellent reversibility. These results indicate that Na enrichment significantly improves cycling stability, and that Zn/Al co-doping further stabilizes the transition-metal layers, suppressing Mn^3+^-induced Jahn-Teller distortions and the irreversible high-voltage phase transition. Taken together, Figure 5b,c show that the Na-rich compositions (Na = 0.80)—NMZA-N14 and NMZA-N10—exhibit much higher long-term capacity retention than the Na = 0.67 counterparts (NM and NMZA-N18). At the same time, NMZA-N18 still outperforms NM, demonstrating that Zn/Al co-doping alone enhances structural stability. The synergistic combination of Na enrichment and Zn/Al co-doping maximizes the stability of both the Na layers and the transition-metal slabs, enabling NMZA-N14 to achieve superior cycling reversibility, rate capability, and structural robustness.

**Figure 6:**
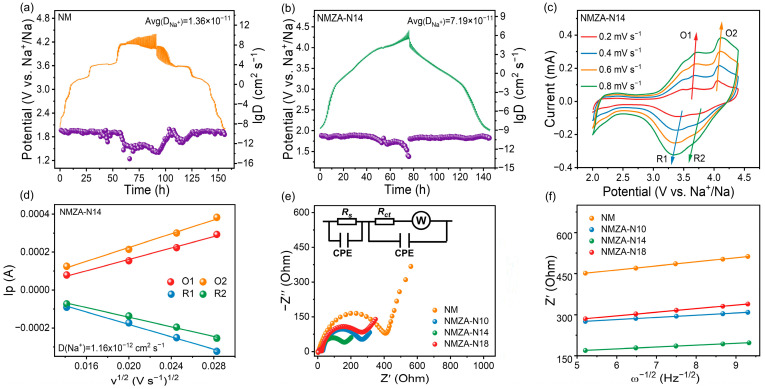
GITT and diffusion analysis for (**a**) NM and (**b**) NMZA-N14; (**c**) CV curves of NMZA-N14 at different scan rates; (**d**) peak current-v^1/2^ relationship for NMZA-N14; (**e**) EIS Nyquist plots of NM, NMZA-N10, NMZA-N14, and NMZA-N18; (**f**) Z′-ω^−1/2^ fitting lines for the same samples.

To assess the effects of Zn/Al co-doping and Na enrichment on Na^+^-transport kinetics, GITT, CV, and EIS measurements were performed on NM, NMZA-N10, NMZA-N14, and NMZA-N18. Figure 6a,b and Figure S5 display the GITT charge–discharge profiles for all samples. The Na^+^ diffusion coefficient (D_Na+_) extracted from GITT indicates that NMZA-N14 reaches 7.19 × 10^−11^ cm^2^ s^−1^, higher than those of NM, NMZA-N10, and NMZA-N18, evidencing faster Na^+^ diffusion kinetics in NMZA-N14. To further probe the reaction kinetics at different scan rates, CV tests were conducted at 0.2, 0.4, 0.6, and 0.8 mV s^−1^ (Figure 6c and Figure S6). According to the Randles-Ševčík relation, the peak current scales linearly with the square root of the scan rate; thus, higher scan rates yield larger peak currents. The NM curves exhibit noticeable peak overlap in the high-voltage region, whereas NMZA-N14 shows a near-linear increase of peak current with v^1/2^, indicating stronger charge-transport capability. The corresponding Na^+^ diffusion coefficient derived from CV is 1.16 × 10^−12^ cm^2^ s^−1^ (Figure 6d), corroborating the Na^+^-diffusion advantage of NMZA-N14. Electrochemical impedance spectroscopy (EIS) was further employed for characterization. In the Nyquist plots (Figure 6e), the high- to mid-frequency semicircle reflects contributions from the cathode-electrolyte interphase resistance (R_s_) and the charge-transfer resistance (R_ct_), while the low-frequency oblique line corresponds to the Warburg impedance (Z_W_) associated with Na^+^ diffusion [32]. According to the fitting results (Table S2), the doped samples exhibit smaller R_ct_ values than the undoped NM. Specifically, R_ct_ for NMZA-N14 is 117 Ω, markedly lower than 351 Ω for NM, indicating that Zn/Al co-doping together with Na enrichment effectively enhances charge-transport kinetics. In addition, Figure 6f presents the Z′-ω^−1/2^ plots, in which the Warburg coefficient (σ) is proportional to the slope. From linear fitting, NMZA-N14 shows a substantially lower σ than NM, NMZA-N10, and NMZA-N18, evidencing a much faster Na^+^ diffusion rate—consistent with its superior rate performance in Figure 5a. Taken together with the GITT and CV analyses, these EIS results demonstrate that NMZA-N14 outperforms NM, NMZA-N10, and NMZA-N18 in Na^+^ transport kinetics; in particular, it combines a higher Na^+^ diffusion rate with a lower charge-transfer resistance, confirming the synergistic benefits of Na enrichment and Zn/Al co-doping on electrochemical performance.

**Figure 7:**
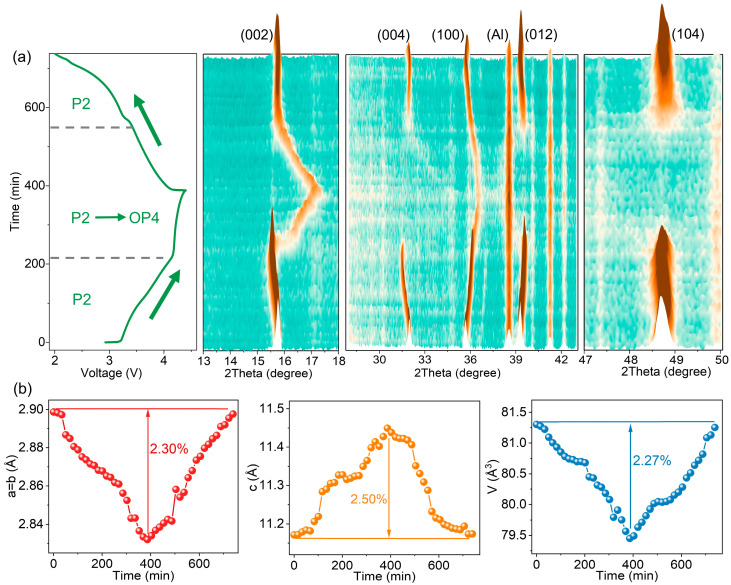
(**a**) In situ XRD patterns of NMZA-N14 at different voltages; (**b**) evolution of lattice parameters (a, b, c) and unit-cell volume during cycling.

To elucidate structural evolution during electrochemical cycling, in situ XRD was carried out on NM and NMZA-N14 (Figure 7 and Figure S7). At the initial stage of charging, both materials show similar diffraction features: the (002) and (004) reflections gradually shift to lower angles, whereas the (100), (012), and (104) peaks monotonically move to higher angles. This indicates a progressive expansion of the unit cell along the c axis with increasing potential, accompanied by in-plane contraction of a/b due to transition-metal oxidation and strengthened interlayer electrostatic interactions [33,34,35]. In the high-voltage region, two-phase coexistence emerges in both cases, but the transformation pathways differ markedly. NM exhibits characteristic reflections of the O2 phase over ~3.8–4.5 V, evidencing a P2 → O2 transition (Figure S7a). By contrast, NMZA-N14 shows OP4 signatures in ~4.1–4.5 V, indicative of a P2 → OP4 transformation (Figure 7a). This divergence highlights the key role of Zn/Al co-doping and Na enrichment in governing the phase-transition mechanism. Refined lattice parameters further resolve the evolution of unit-cell volume (Figure 7b and Figure S7b). For NMZA-N14, the lattice constants adjust reversibly during cycling: a(b) contracts by 2.30%, c expands by 2.50%, and the overall unit-cell volume fluctuates by only 2.27%. In contrast, NM exhibits changes of 5.04% in a(b) and 14.25% in c, resulting in a unit-cell volume shrinkage as large as 6.28%. Moreover, upon discharge, the OP4 reflections of NMZA-N14 nearly disappear and the P2 structure is restored, demonstrating high reversibility. Overall, Zn/Al co-doping together with Na enrichment suppresses the high-voltage P2 → O2 transition and redirects the system toward a P2 → OP4 pathway with smaller volume effects and higher reversibility. This structural regulation not only mitigates lattice-strain accumulation but also provides a crystallographic basis for the superior cycling stability and rate performance.

## 3. Materials and Methods

### 3.1. Preparation of Materials

Four layered sodium-ion cathode materials were prepared: Na_0.67_Ni_0.33_Mn_0.67_O_2_ (denoted NM), Na_0.80_Ni_0.10_Zn_0.18_Mn_0.58_Al_0.14_O_2_ (NMZA-N10), Na_0.80_Ni_0.14_Zn_0.14_Mn_0.58_Al_0.14_O_2_ (NMZA-N14), and Na_0.67_Ni_0.18_Zn_0.10_Mn_0.58_Al_0.14_O_2_ (NMZA-N18). All samples were synthesized via a sol-gel route. The starting reagents included sodium acetate (NaAc, 99.0%, Innochem, Beijing, China; an additional 5% was used to compensate for Na loss during high-temperature calcination), Mn(CH_3_COO)_2_·4H_2_O (99.99%, Aladdin, Shanghai, China), Ni(CH_3_COO)_2_·4H_2_O (99.99%, Aladdin), Al_2_(NO_3_)_3_·9H_2_O (99.99%, Aladdin), and Zn(CH_3_COO)_2_·4H_2_O (99.8%, Aladdin). Citric acid (99.99%, Shanghai Reagent, Shanghai, China) was employed as the complexing agent.

Metal salts and NaAc were weighed according to the target stoichiometries and dissolved in deionized water under magnetic stirring. Citric acid was then introduced and stirring continued to afford a homogeneous precursor solution. The solution was heated and stirred at 80 °C until its viscosity increased and a stable sol formed. The sol was dried in an oven at 120 °C for 12 h to yield a xerogel precursor. The dried gel was ground into a fine powder and calcined at 450 °C for 5 h in a tube furnace to remove organics and residual ligands. After cooling to room temperature, the powder was reground and subsequently calcined in air at 850 °C for 18 h to obtain the final layered sodium transition-metal oxides. The products were naturally cooled to room temperature, collected, and used for subsequent structural characterization and electrochemical measurements.

### 3.2. Material Characterization

The crystal structures of the samples were characterized by X-ray diffraction (XRD, Cu Kα radiation; λ_1_ = 1.5406 Å, λ_2_ = 1.5444 Å) over a 2θ range of 10–90°. The diffraction profiles were subjected to Rietveld refinement using GSAS-II (Version 5455) to obtain lattice parameters and structural information. The microstructures and particle-size distributions were examined by scanning electron microscopy (SEM, Regulus 8100, Hitachi, Tokyo, Japan), while transmission electron microscopy (TEM, JEM-2100F, JEOL, Tokyo, Japan) was employed to resolve lattice fringes and selected-area electron diffraction (SAED) patterns, further corroborating the crystallographic features. X-ray photoelectron spectroscopy (XPS, K-Alpha, Thermo Fisher Scientific, Waltham, MA, USA) was employed to analyze the surface elemental composition and to determine the oxidation states and chemical environments of the constituent elements. The XPS measurements were conducted using an Al Kα X-ray source (hν = 1486.6 eV; λ ≈ 0.834 nm), and all binding energies were calibrated to the adventitious carbon C 1s peak at 284.8 eV.

### 3.3. Electrochemical Measurements

Working electrodes were formulated with 70 wt% active material, 20 wt% acetylene black (conductive additive), and 10 wt% poly(vinylidene fluoride) (PVDF, binder). PVDF was first dissolved in N-methyl-2-pyrrolidone (NMP) to prepare the binder solution; the active material and acetylene black were then added and dispersed under magnetic stirring to obtain a homogeneous slurry. The slurry was uniformly coated onto aluminum-foil current collectors and dried under vacuum at 120 °C for 12 h. The resulting electrode film had a thickness of ~200 μm (controlled by a doctor blade), with an active-material mass loading of ~3.5–3.8 mg cm^−2^. The dried electrodes were subsequently punched into 12-mm-diameter discs for use as working electrodes. The dried electrodes were punched into discs and used as working electrodes. For half-cell assembly, sodium metal served as both counter and reference electrodes, a glass-fiber separator (Whatman GF/D, Canrd Technology Co., Ltd., Dongguan, China) was employed, and the electrolyte consisted of 1 M NaPF_6_ dissolved in diglyme (Canrd Technology Co., Ltd., Dongguan, China).

Galvanostatic charge-discharge (GCD) tests were carried out on a Land CT-2001A system within a voltage window of 2.0–4.4 V. Galvanostatic intermittent titration technique (GITT) measurements were performed on the same system using a protocol of 0.1C current (1C = 173 mAh g^−1^) applied for 10 min followed by a 40 min relaxation, to determine the Na^+^ diffusion coefficient. Cyclic voltammetry (CV) was conducted on a CHI660-E electrochemical workstation over 2.0–4.4 V at a scan rate of 0.1 mV s^−1^ to analyze redox characteristics and reaction reversibility. Electrochemical impedance spectroscopy (EIS) was performed on a Zahner Ennium workstation over 0.01 Hz–100 kHz with an AC amplitude of 5 mV to evaluate charge-transfer resistance and ion-diffusion behavior.

## 4. Conclusions

Through the synergistic regulation of Zn/Al co-doping and Na enrichment, we successfully synthesized a P2-Na_0.80_Ni_0.14_Zn_0.14_Mn_0.58_Al_0.14_O_2_ cathode and systematically elucidated the origins of its enhanced structural stability and electrochemical performance. The optimized material delivers an initial discharge capacity of 125 mAh g^−1^ at 0.1C and achieves remarkable capacity retention under both long-term cycling and high-rate operation—98.6% after 100 cycles at 0.2C and 93.6% after 200 cycles at 1C—highlighting its excellent cycling stability and rate capability. Kinetic analyses further confirm a higher Na^+^ diffusion coefficient and lower charge-transfer resistance, indicating markedly improved ion transport and interfacial reaction kinetics. In situ XRD reveals a reversible P2 → OP4 transition in the high-voltage regime with a unit-cell volume fluctuation of only 2.27%, effectively avoiding the irreversible P2 → O2 transformation and associated structural degradation typical of the benchmark NM material. Overall, the cooperative effects of Zn/Al substitution and Na enrichment stabilize both the transition-metal slabs and the Na layers, offering a viable design principle for layered sodium-ion cathodes that simultaneously achieve high stability and high performance.

## Data Availability

The original contributions presented in this study are included in the article/Supplementary Material. Further inquiries can be directed towards the corresponding authors.

## Supplementary Materials

The following supporting information can be downloaded at https://www.mdpi.com/article/10.3390/molecules30234628/s1, Figure S1: Rietveld refinement profiles of the XRD patterns for (a) NM, (b) NMZA-N10, and (c) NMZA-N18; Figure S2: EDS elemental mapping of NMZA-N14; Figure S3: The Mn 3s sperctrum of (a) NM and (b) NMZA-N14; Figure S4: Charge-discharge curves of (a) NMZA-N10 and (b) NMZA-N18; Figure S5: GITT charge-discharge profiles and diffusion-coefficient calculations for (a) NMZA-N10 and (b) NMZA-N18; Figure S6: CV curves of NM at different scan rates; Figure S7: In situ XRD patterns and refined lattice parameters of NM; Table S1: Lattice parameters and R-factors of NM, NMZA-N10, NMZA-N14, and NMZA-N18 obtained from the Rietveld refinement; Table S2: EIS fitting parameters for NM, NMZA-N10, NMZA-N14, and NMZA-N18.
