# Supplementary material for: Synergistic Zn/Al Co-Doping and Sodium Enrichment Enable Reversible Phase Transitions in High-Performance Layered Sodium Cathodes
Source: Molecules. 2025 Dec 2;30(23):4628. doi: 10.3390/molecules30234628 (PMC12692805; doi:10.3390/molecules30234628)
Supplement: Supplementary file 1 [file molecules-30-04628-s001.zip › molecules-4010181-Supplementary.pdf]

Supporting Information

Synergistic Zn/Al Co-Doping and Sodium Enrichment Enable Reversible Phase Transitions in High-Performance Layered Sodium Cathodes

Yaru Qin[1,2,†], Tingfei Yang[1,3,†], Na Chen[3], Jiale Li[1], Anqi Li[3], Yu Miao[1], Chenglong Shi[1,2,*], Jianmin Ma[1,*], Xue Qin[3,*]

[1] School of Chemistry and Materials Science, Qinghai Minzu University, Xining 810007, China

[2] Key Laboratory of Resource Chemistry and Eco-Environmental Protection on Tibetan Plateau, State Ethnic Affairs Commission, Qinghai Minzu University, Xining 810007, China

[3] Department of Chemistry, School of Science, Tianjin University, Tianjin 300072, China

* Correspondence: shiclongcas@163.com (C.S.); majminmz@126.com (J.M.); qinxue@tju.edu.cn (X.Q.); Tel.: +86-09718173864 (C.S. & J.M.); +86-02227406577 (X.Q.)

† These authors contributed equally to this work.

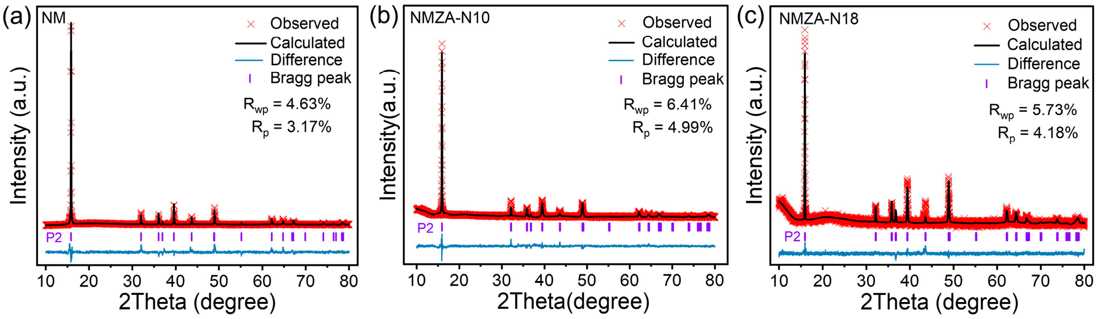

Figure S1. Rietveld refinement profiles of the XRD patterns for (a) NM, (b) NMZA-N10, and (c) NMZA-N18.

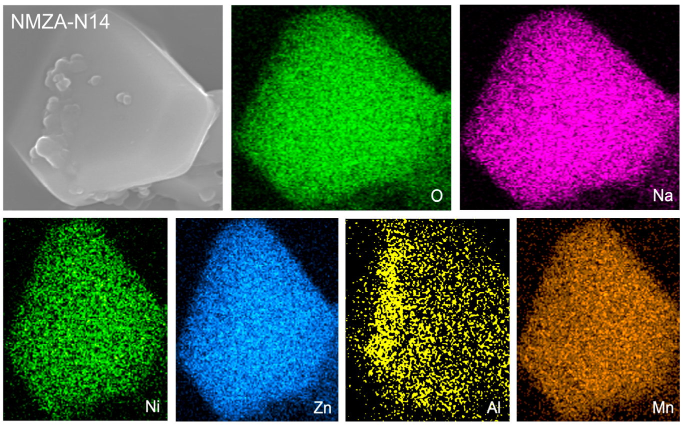

Figure S2. EDS elemental mapping of NMZA-N14.

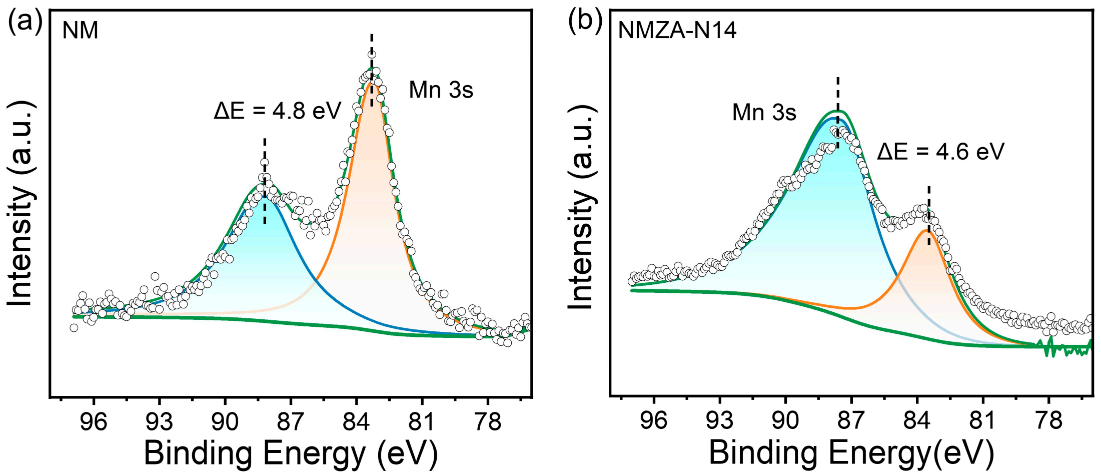

Figure S3. The Mn 3s sperctrum of (a) NM and (b) NMZA-N14.

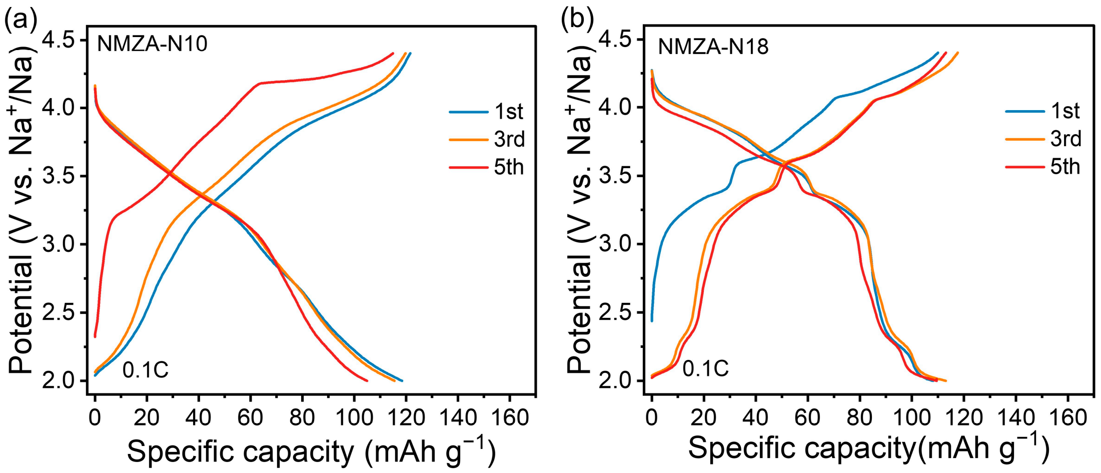

Figure S4. Charge-discharge curves of (a) NMZA-N10 and (b) NMZA-N18.

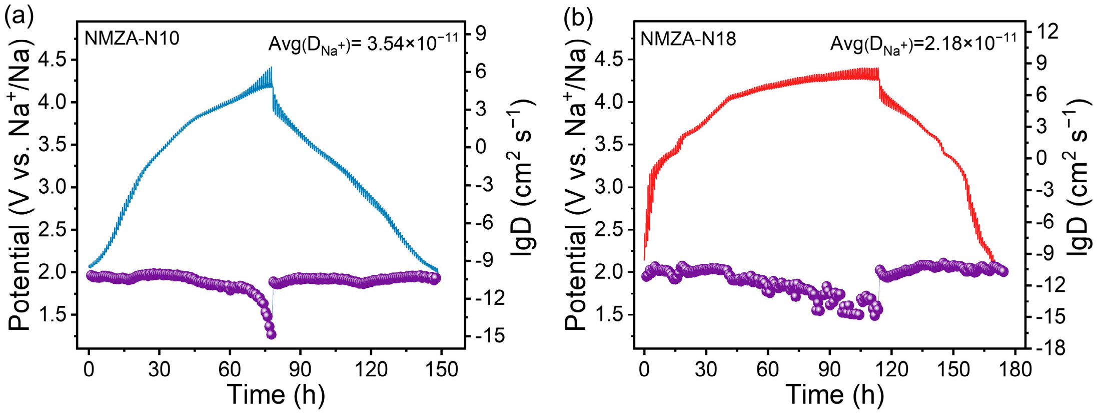

Figure S5. GITT charge－discharge profiles and diffusion-coefficient calculations for (a) NMZA-N10 and (b) NMZA-N18.

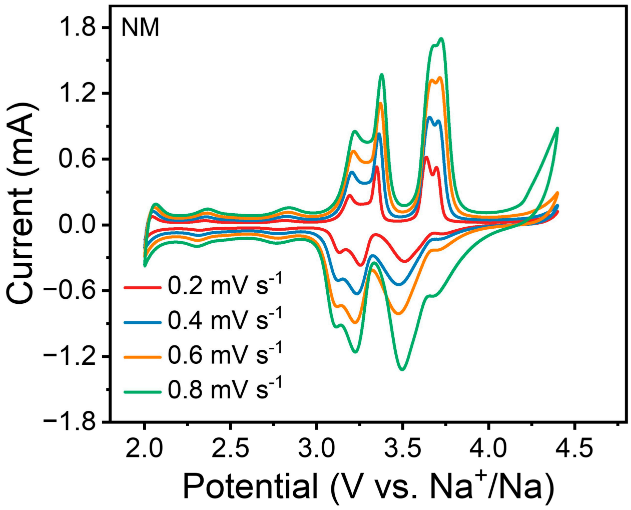

Figure S6. CV curves of NM at different scan rates.

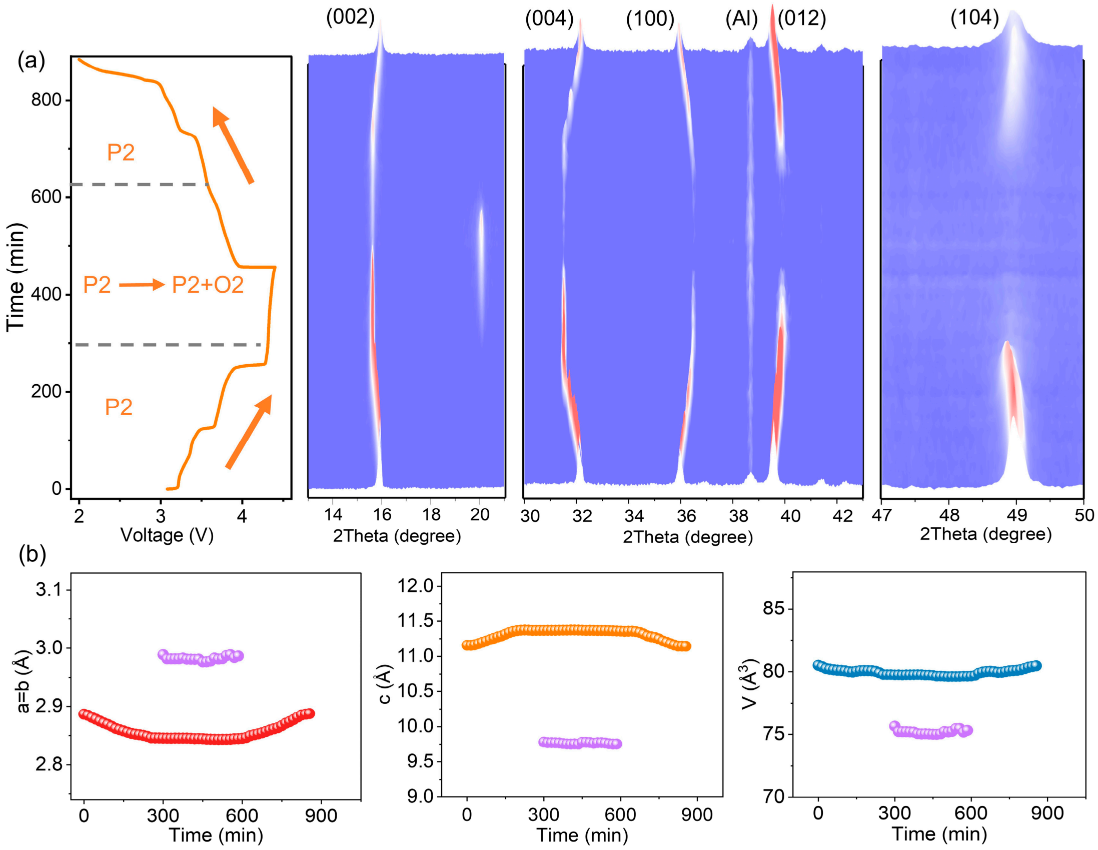

Figure S7. In situ XRD patterns and refined lattice parameters of NM.

Table S1 Lattice parameters and R-factors of NM, NMZA-N10, NMZA-N14, and NMZA-N18 obtained from the Rietveld refinement.

Sample	a (Å)	b (Å)	c (Å)	V (Å^3)	R_{wp}	R_p
NM	2.875	2.875	11.184	80.07	4.63%	3.17%
NMZA-N10	2.892	2.892	11.146	80.71	6.41%	4.99%
NMZA-N14	2.892	2.892	11.135	80.65	4.74%	3.53%
NMZA-N18	2.895	2.895	11.143	80.88	5.73%	4.18%

Table S2. EIS fitting parameters for NM, NMZA-N10, NMZA-N14, and NMZA-N18.

Sample	R_s (Ω cm^{-2})	R_{ct} (Ω cm^{-2})	Fitting error (%)	σ (Ω s$^{-1/2}$)
NM	28.4	351	2.91	14.7
NMZA-N10	27.1	221	2.73	8.1
NMZA-N14	21.6	117	4.46	6.7
NMZA-N18	21.3	232	4.16	13.1
